# Gas Pulse–X-Ray Probe Ambient Pressure Photoelectron Spectroscopy with Submillisecond Time Resolution

**DOI:** 10.1021/acsami.1c13590

**Published:** 2021-09-30

**Authors:** Andrey Shavorskiy, Giulio D’Acunto, Virginia Boix de la Cruz, Mattia Scardamaglia, Suyun Zhu, Robert H. Temperton, Joachim Schnadt, Jan Knudsen

**Affiliations:** † MAX IV Laboratory, Lund University, Lund 221 00, Sweden; ‡ Division of Synchrotron Radiation, Department of Physics, Lund University, Lund 221 00, Sweden

**Keywords:** APXPS, CO oxidation, time-resolved XPS, catalysis, operando spectroscopy

## Abstract

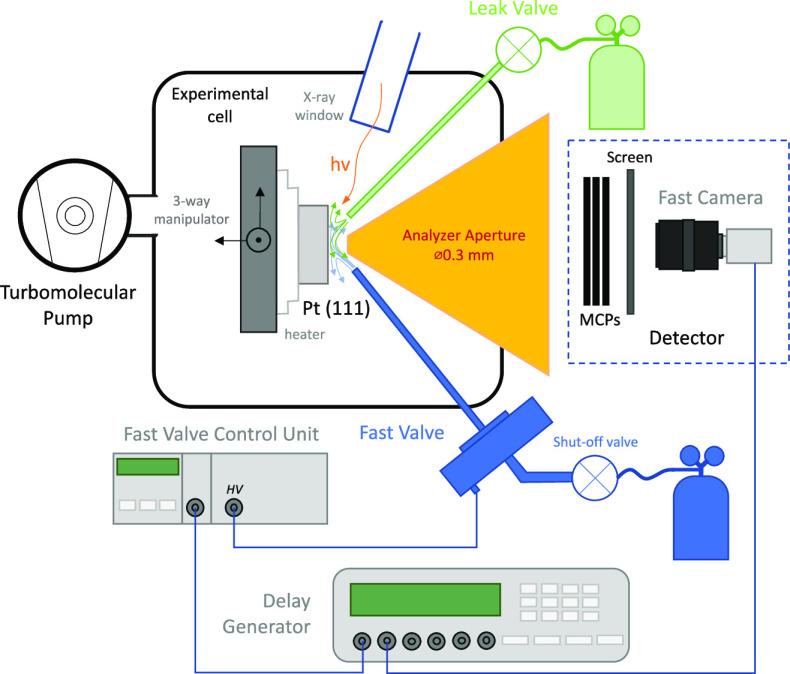

A setup capable of conducting gas pulse–X-ray probe ambient pressure photoelectron spectroscopy with high time resolution is presented. The setup makes use of a fast valve that creates gas pulses with an internal pressure in the mbar range and a rising edge of few hundreds of microseconds. A gated detector based on a fast camera is synchronized with the valve operation to measure X-ray photoemission spectra with up to 20 μs time resolution. The setup is characterized in several experiments in which the N_2_ gas is pulsed either into vacuum or a constant flow of another gas. The observed width of the pulse rising edge is 80 μs, and the maximum internal pulse pressure is ∼1 mbar. The CO oxidation reaction over Pt (111) was used to demonstrate the capability of the setup to correlate the gas phase composition with that of the surface during transient supply of CO gas into an O_2_ stream. Thus, formation of both chemisorbed and oxide oxygen species was observed prior to CO gas perturbation. Also, the data indicated that both the Langmuir–Hinshelwood and Mars-van-Krevelen mechanisms play an important role in the oxidation of carbon monoxide under ambient conditions.

## 1 Introduction

Chemical transformations occurring at the interfaces between gases and solids are the driving forces responsible for multiple industry-relevant processes such as heterogeneous catalysis, gas sensing, and thin film growth. These processes are dynamic by nature with various steps occurring at different time scales.^1^ For instance, it takes a few nanoseconds for an elementary catalytic step to occur on a surface of a catalyst, whereas it takes much longer time—milliseconds to seconds—for morphological changes such as segregation, solid-state diffusion, or particle restructuring to occur.^2^ Hence, to obtain a complete picture about surface phenomena one has to study their time evolution using time-sensitive experimental techniques. Accordingly, such techniques must access timescales that are characteristic of the specific process and system of interest. However, it is often meaningless to simply reach for the highest possible time resolution when studying systems under equilibrium or steady-state conditions: even though dynamical processes do occur continuously, their equilibrium nature implies that they are masked in the spectroscopic data because of the simultaneous occurrence of the corresponding back reactions. It is only when the system is driven away from equilibrium its time evolution becomes truly observable. Thus, the general scheme for a time-resolved technique is as follows: a system previously in the equilibrium/steady-state experiences a perturbation of some nature, which drives it into a nonequilibrium state. The system is then allowed to relax either to the previous equilibrium (for reversible processes) or a new equilibrium (for nonreversible processes); meanwhile, it can be investigated by a time-resolved experimental method. Both the excitation and measurement should normally occur at much shorter timescales than the subsequent relaxation for the experiment to provide meaningful data.

A system can be perturbed by a variety of methods (which is the “pump” step of the experiment); the most commonly used pumps are optical. The system’s response can then be monitored, for example, using the time structure of an X-ray source, X-ray/electron detectors, or their combination (this is the “probe” step of the experiment); time-resolved information is obtained by either time recording/tagging or gating/discriminating schemes. In the first scheme, a timestamp is recorded for each individual event with respect to some global reference (e.g., the internal clock of a computer or the detector turn-on time). In the latter scheme, the measurement of events is synchronized with a periodic reference signal (normally the perturbation itself) such that only events at a specific delay are recorded.

With respect to the exploration of surface phenomena, X-ray photoelectron spectroscopy (XPS) is an advantageous method as it is highly surface-sensitive, chemically specific, and quantitative. Time-resolved studies of surface processes based on the use of XPS have been very fruitful for obtaining an understanding of a wide range of surface chemical processes.^3−7^ Synchrotron-based time-resolved (tr) XPS experiments which focus on the dynamics of the surface photovoltage on semiconductor and metal oxide surfaces^8^ or the charge transfer between metal–organic complexes and metals/semiconductor support materials^9^ can now routinely achieve a time resolution that ranges from tens of picoseconds at modern electron storage rings^10,11^ to femtoseconds on free-electron lasers^12^ or high-harmonic generation light sources.^13^ Such studies primarily make use of an optical perturbation as a pump. tr-XPS experiments that rely on pressure or chemical perturbations have so far not implemented a pump–probe scheme. This limits their time resolution to a minimum time necessary to record a single photoemission spectrum with a satisfactory signal-to-noise ratio, typically several hundred milliseconds at modern synchrotron light sources.^7,6,14,15^ Using much shorter acquisition times, such as a few milliseconds, it is normally not possible to acquire a single spectrum with good enough statistics. Thus, to reach an improved time resolution and yet maintain a satisfactory signal-to-noise ratio, the measurement must be repeated multiple times, and the collected data must then be averaged to improve the quality of the data. Such averaging could be performed either using an external or internal reference signal. The last one is advantageous in cases of irregularities in the gas pulses or the surface response to the pulses and can also be used for event-average pulses created by self-sustained reaction oscillations. This requires, however, that the investigated process be reversible and repetitive.

Using internal reference signals found by image recognition, Knudsen et al.^4^ demonstrated event averaging over many ambient-pressure X-ray photoelectron spectroscopy (APXPS) pulse/probe cycles such that time-resolved X-ray photoelectron (XP) spectra with otherwise poor statistics could be improved to obtain a sufficient signal-to-noise ratio, without losing time resolution. By searching for a lock-in signal in the data itself, the method allows for averaging data originating from nonstrictly periodic reactions or experiments with significant time deviations from supposedly periodic events (e.g., valve opening, gas flow, or spectra collection). This enabled them to observe the transient-gas supply-oscillation of a Pd(100) surface between the phases active and inactive for the CO oxidation reaction with 60 ms time resolution. However, it still requires a sufficient signal-to-noise ratio to find a lock-in signal by the image recognition algorithm.

The best measured time resolution in a gas pulse pump–probe tr-XPS, 500 μs, using an external reference was achieved by Höfert et. al^16^ in an adsorption experiment of CO on Pt(111). In the experiment, gas was introduced into the sample using a supersonic molecular beam at a pressure of 2 × 10^–6^ mbar for a well-defined period of time, and the surface composition was measured using a fast detector. Despite excellent time resolution, the maximum pressure of the gas pulse was still six orders of magnitude lower than that normally used in a typical APXPS measurement.

The interaction of carbon monoxide and oxygen with Pt-group metals is one of the industry-relevant processes that can particularly benefit from experiments with high time resolution because of high turnover frequency of the CO_2_ production reaction.^17^ This reaction is extremely well studied under ultrahigh vacuum (UHV) conditions. There is a widely accepted agreement that the metallic phase is an active phase and that, above a critical temperature and when the surface becomes CO-free, the CO oxidation reaction occurs via the Langmuir–Hinshelwood (LH) mechanism. Under these conditions, the active species are oxygen atoms that are formed via O_2_ dissociation and get rapidly consumed by adsorbed CO molecules to form CO_2_.^18^

Things seem to get more complicated when the reactants’ pressure rises already to around a mbar. Although there exists a large body of research from the last two decades on the Pt-based CO oxidation reaction under these conditions, there is no commonly accepted picture on the active catalytic phase, chemical state of the metal surface, or reaction mechanism. There are studies that strongly support the notion of a LH mechanism with the metallic platinum as the active phase.^19−24^ At the same time, other studies state evidence for the formation of a platinum surface oxide with a very high catalytic activity toward CO oxidation via the Mars-van-Krevelen (MK) mechanism.^25−29^ One reason for the lack of agreement about formation of the oxide and its role could be its transient nature: the oxide can be formed only within a narrow window of experimental conditions when the surface is most active and will disappear when the reaction rate decreases, that is, in the mass-transfer limit (MLT). The high-time-resolution measurements of the surface and gas composition will make it possible to follow the evolution of the surface oxide and correlate it with the catalytic activity of the system.

Introducing ambient conditions to tr-XPS (that is performing time-resolved ambient-pressure XPS or tr-APXPS) can, on the one hand, be very fruitful because of the possibility of resolving surface dynamics in real systems and under real conditions.^14^ On the other hand, as it was demonstrated in the previous literature, tr-APXPS experiments become complicated by the additional drop in the count rate because of the scattering of electrons by the gas phase at elevated pressures.^4,15,16,30^ Additional challenge for tr-APXPS with rapid pressure/chemical perturbations is needed to generate gas pulses with time constants that are comparable to those of the time resolution of the experiment. Such gas pulse perturbations can be achieved through gas flow control by mass flow controllers (MFCs)^15^ or using supersonic molecular beams (SSMBs).^16^ MFCs have the advantage of precise flow/pressure control and high reproducibility, but currently they are somewhat too slow for microsecond tr-APXPS; today, the fastest MFCs for vacuum applications have a response time in the range of 50–100 ms.^31^ SSMB setups can routinely achieve 50 μs pulses, but the maximum pressure in the pulse is several orders lower than 1 mbar.^32^ The use of fast valves for generating ms-long (with sub-millisecond rising edge) gas pulses with pulse pressure up to tens of mbars was demonstrated by Amati et al.^33^ Authors employed a fast pulsed valve and a nozzle to create dynamic high pressure in front of the sample to overcome incompatibility of their UHV XPS machine to ambient conditions. For example, 3.2 ms pulses of O_2_ injected at 0.35 Hz rate were equivalent to a static oxygen pressure between 10^–3^ and 10^–2^ mbar, which was three orders of magnitude higher than the highest operational pressure in the setup (∼10^–5^ mbar). The background pressure in the chamber, on the other hand, remained below highest operational pressure allowing in situ measurement of XPS even under UHV conditions. However, tr-XPS measurements with similar time resolution were not demonstrated because of the absence of a time-sensitive detector.

This paper describes a setup capable of measuring tr-APXPS with gas pressure perturbations with 20 μs time resolution. The setup uses a fast valve to create gas pulses with an internal pressure in the mbar range and rising edge of few hundreds of microseconds. The action of the valve is synchronized to the opening of the shutter on the detector’s fast camera through the delay generator. We characterize the gas pulse in a single-gas experiment where the signal of the N_2_ gas phase is studied versus the attenuation of the sample’s signal. Then, with a double-gas experiment we investigate the behavior of an N_2_ gas pulse in a background of CO_2_. Finally, the oxidation of CO over Pt (111) was used to demonstrate the capabilities of the setup by correlating the gas phase and surface composition during the first 200 μs of transient supply of CO gas into an O_2_ stream.

## 2 Experimental Setup

The experiments were performed at the HIPPIE beamline at the Swedish national synchrotron radiation facility MAX IV Laboratory in Lund, Sweden.^15^ The APXPS endstation is equipped with a Scienta Omicron HiPP-3 electron energy analyzer capable of measuring XP spectra at pressures up to 30 mbar. All measurements were conducted inside the “EC cell” setup chamber with a background pressure of 1 × 10^–5^ mbar and ∼70 L inner volume. A Pt(111) single crystal sample was mounted on a flag-style sample holder and cleaned with Ar sputtering (1 kV, 5 mA) for 15 min in the preparation chamber at the HIPPIE endstation. After cleaning, it was transferred through the atmosphere into the EC cell and fixed onto the cell’s three-way manipulator. The sample was heated using a ceramic heater with carbon electrodes up to 500 °C. Materials that were subjected to high temperature are stainless steel, alumina, and BN ceramics. This ensured that no catalytic reaction could occur on any parts of the setup other than on the platinum sample itself. After mounting the sample, the EC cell was pumped down to 1.0 × 10^–5^ mbar. Survey spectra were taken to assess the cleanness of the sample. Only residual carbon was detected on all occasions; prior to the pulsed experiments, the carbon contamination was burned away at elevated temperature in 1.0 × 10^–2^ mbar of O_2_. The pressure inside the experimental volume was measured using a Pfeiffer PKR360 full-range gauge. This pressure measurement represents the average pressure in the experimental chamber because of the large volume of the chamber and the slow readout time of the gauge that was not fast enough to react to the ms gas pulse.

### 2.1 Creation of Short Pulses

**Figure 1:**
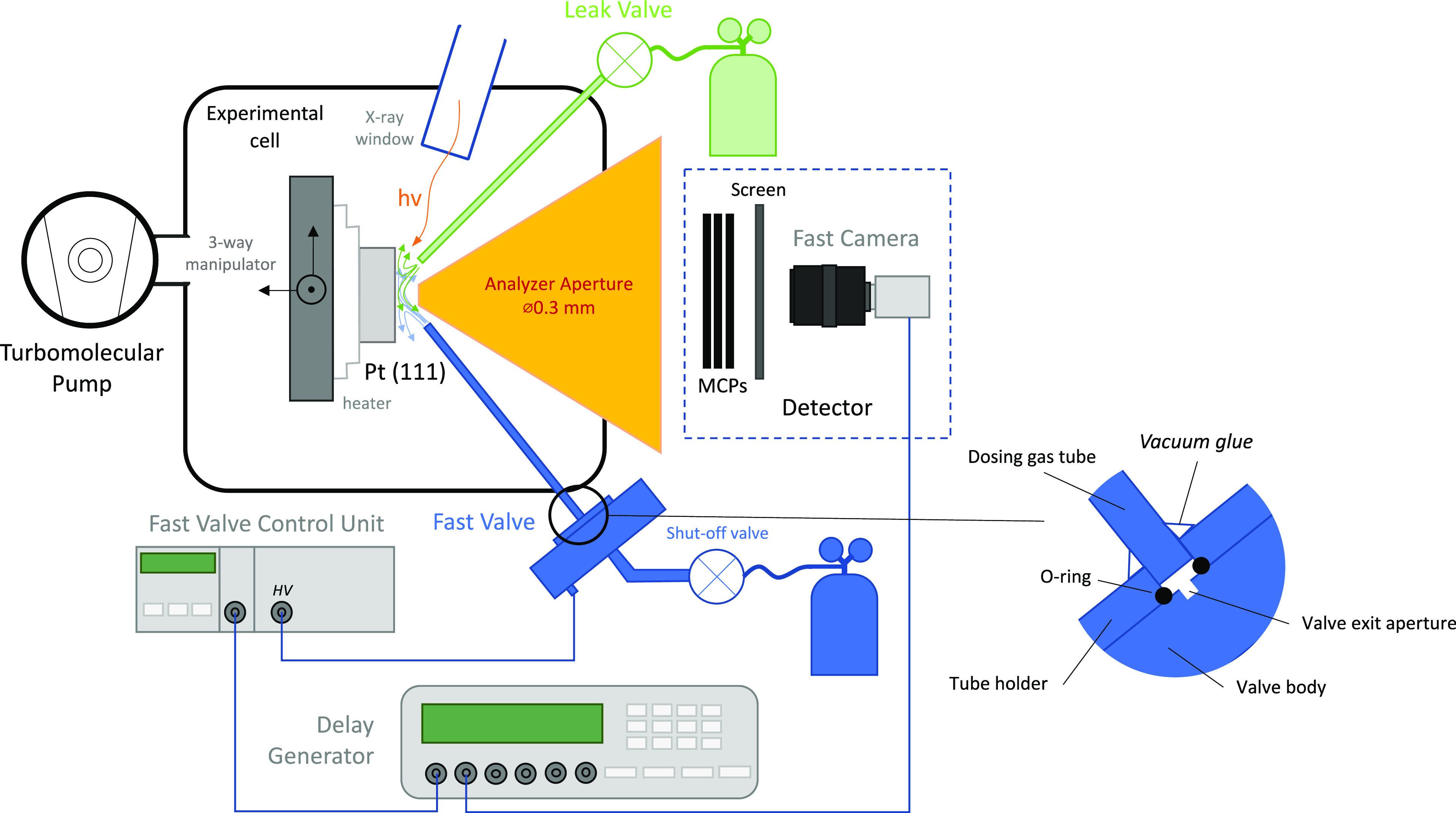
Experimental setup for time-resolved APXPS gas pulse experiments. Note that the fast valve can be placed both inside and outside the experimental cell. See text for details of the setup.

The scheme of the setup for creating fast pulses of gas with mbar internal pressure is shown in Figure 1. An Attotech GR020 piezo valve is used to generate short pulses of gas.^34^ The valve body can be placed inside or outside of the experimental cell and is connected to an Attotech DU001 drive unit with a high voltage cable. The drive unit provides power to the piezo actuator inside the valve and is controlled using an external trigger signal that comes from a BNC 565 signal/delay generator. The combination allows full control over the valve operation by control of the high voltage, frequency, and duration of the opening as well as synchronization with the detector. The valve is cooled with 25 °C water to protect the piezo mechanism and ensure constant temperature. For the valve to open, the voltage applied to the piezo actuator needs to surpass a threshold value of ∼160 V. The absolute value of the voltage defines the size of valve opening and thus the pressure within the gas pulse: a higher voltage corresponds to a higher pressure. The high-pressure side of the valve is connected to the cylinder that contains the gas of which pulses should be produced. The pressure in the gas-providing line is held at around 2–4 bar absolute pressure using a dual-stage regulator. It needs to be noted that the pressure of the gas on the high-pressure side of the valve is a parameter that affects the pressure inside pulse and that it therefore needs to be controlled. At the low-pressure side of the valve, a pinhole 1 mm in diameter was drilled in the valve body. A straight 0.6 mm ID tube was then tightly attached to the pinhole to create a confined space for the directional propagation of the gas pulse. The tube ending is positioned a few mm away from the sample surface.

In a two-gas experiment, the second gas is constantly dosed onto the sample’s surface from a second tube of the same diameter as the above described gas supply tube. It is also placed at a similar distance from the surface. The second tube is connected to a leak valve. The constant flow of the second gas is needed to ensure complete return to equilibrium after driving the sample away from it by the pulse of the first gas.

The experimental cell is constantly pumped using a Pfeiffer HiPace 300 turbopump to ensure quick gas removal.

### 2.2 Time-Resolved Detection of Gas and Surface Evolution

**Figure 2:**
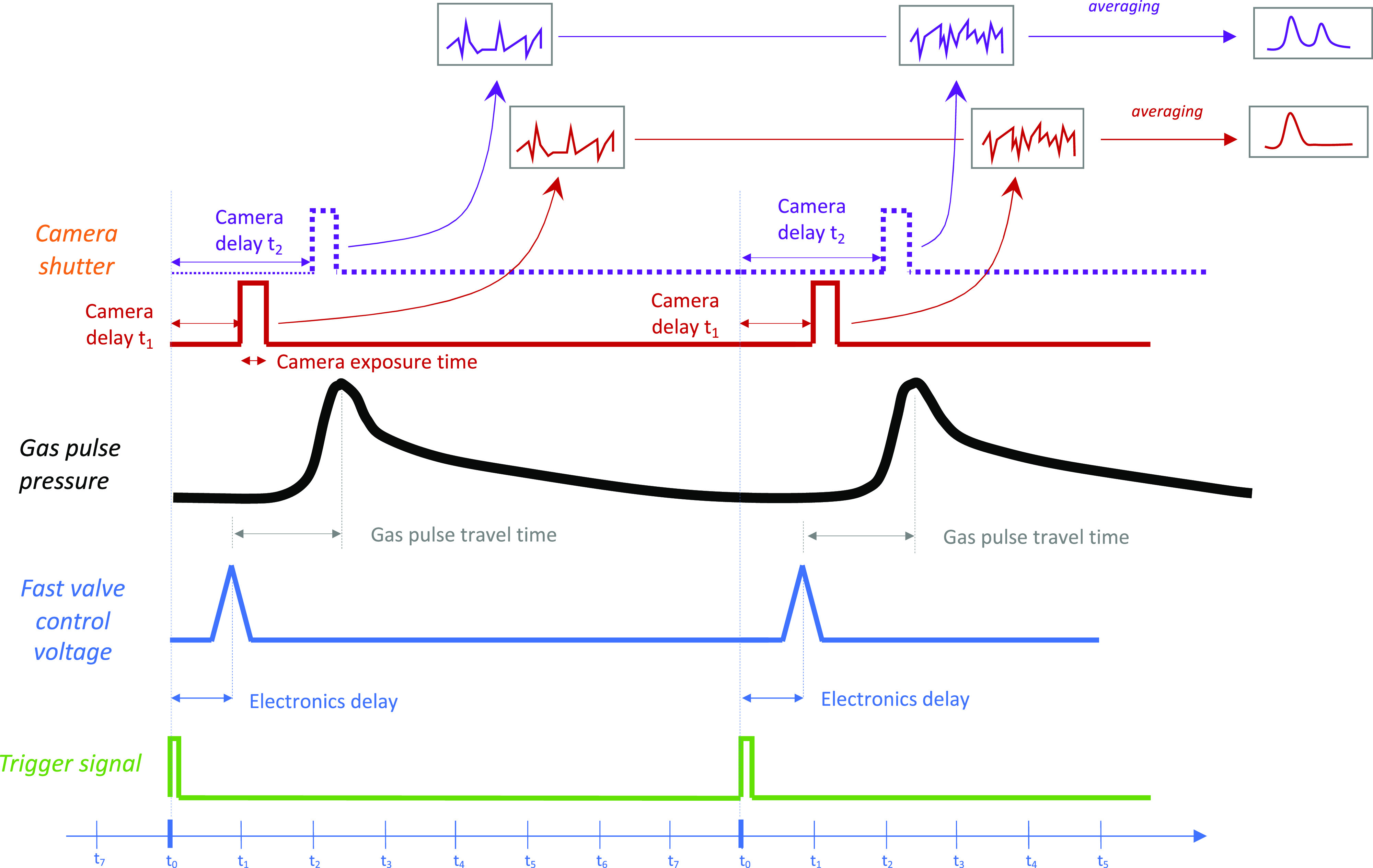
Time diagram for time-resolved (AP)XPS. See text for a detailed explanation.

Time-resolved experiments were conducted using the time gating scheme with the detector being synchronized with the piezo valve through the delay generator (Figure 1). The detector—a 120 Hz Basler camera—is equipped with a fast shutter that allows exposures down to 20 μs. The opening of the shutter is controlled by a trigger signal from the delay generator. Exposure times used in below discussed time-resolved experiments varied from 20 to 80 μs, a compromise reflecting the trade-off between the length of the experiments, required for collection of satisfactory signal-to-noise ratios (which requires longer exposure time), and maximizing time resolution (requiring lowering exposure time). Figure 2 shows the time-synchronization scheme for the described experiments. At the bottom of the figure, the periodic trigger signal (which defines time t_0_) from the delay generator is shown as a green line. This is a 0–5 V signal with a fast (<5 ns rising time) edge. Directly above it, the signal that controls the opening of the fast valve is depicted as a blue line. Note that the short delay between the trigger signal and opening of the valve is due to a delay in the processing of the trigger signal by the delay generator. As described above, opening of the valve results in a gas leak through the valve and dosing tube onto the sample, which is schematically shown in Figure 2 as a black line. The delay between the valve opening and the gas pulse reaching the sample is due to the time it takes for the gas pulse to travel through the tube. The long length of the gas pulse, compared to the valve opening time, is likely due to several factors including scattering of the gas molecules from the walls of the tube and the limited pumping rate. Finally, red and purple lines show the operation of the camera shutter for several time delays. Because of the fixed delay between the valve opening and camera shutter operation during each pulse, the data are collected from the same part of the pulse. Thus, it becomes possible to average multiple spectra with the same time delay, and event-averaged spectra acquired at specific times within the pulse can be constructed (shown at the top of Figure 2). By performing similar measurements at multiple delays, a time-dependent evolution of the photoelectron spectrum can be recorded for the whole gas pulse.

Special care needs to be taken to ensure that the count rate is always in the linear range of the detector response: at high count rates, the microchannels do not have enough time to discharge before they are hit by another electron, and the signal becomes nonlinear with the intensity. This problem is apparent and easy to monitor in the non-time-resolved mode because all electrons that hit the detector are detected by the camera. However, in the time gating mode only a portion of electrons that arrive on the detector are within the exposure window and thus detected by the camera. At 40 μs exposure time and a 25 Hz repetition rate, 99.9% of the electrons are outside of the exposure window. In our experiments, the beamline and analyzer settings were optimized to maximize the count rate for a given region in the non-time-resolved mode. After that, a time-resolved spectrum was taken with the desired exposure time and repetition rate, and the spectra were acquired until a satisfactory signal-to-noise ratio was achieved.

### 2.3 Details of the Experiments

In the experiments described below, the XPS measurements were conducted with the sample in one of the three positions: the XPS position, where the intensity of the surface signal is at maximum (∼300 μm away from the analyzer aperture) while the gas phase intensity is low; the gas phase position, with the sample retracted so that no electrons from the surface could be detected (∼600 μm away from the analyzer aperture); and the hybrid position, where the measurement of both the gas phase and surface signals is balanced to collect sufficient statistics in a reasonable amount of time without moving samples in between measurements (450–500 μm away from the analyzer aperture). In the gas phase position, the beamline slit could normally be increased to raise the count rate. This led to shorter measurement times for the gas phase spectra. The analyzer aperture size was 300 μm.

All N 1s and O 1s spectra were measured with 750 eV excitation energy. C 1s and Pt 4f spectra were measured with 500 and 350 eV excitation energies, respectively. The XPS data were analyzed in the IgorPro software. Linear or Shirley backgrounds were subtracted prior to curve-fitting the spectra. Symmetric pseudo-Voigt peak shapes were used for fitting of C 1s, N 1s, and O 1s spectra, whereas asymmetric pseudo-Voigt shapes were used for the Pt 4f line. Pt 4f_7/2_ spectra in Figure 6 could not be fitted because of the incomplete region which prevented subtraction of the correct Shirley background. Recording the complete Pt 4f spectrum would require doubling measurement time which was not feasible for the presented experiments. However, the qualitative analysis of the spectral shapes presented for the CO oxidation on the Pt(111) section (Figure 6) is totally adequate to support findings presented in the paper.

Because of the limited experimental time and the time-gated acquisition scheme, only delays of the highest interest were measured. These data points generally lie within the pulse rising edge or just before it. This is the reason for variation in the data point densities in some of the following examples (e.g., Figure 5).

## 3 Results

### 3.1 Pulse Characterization: Single-Gas Experiment

**Figure 3:**
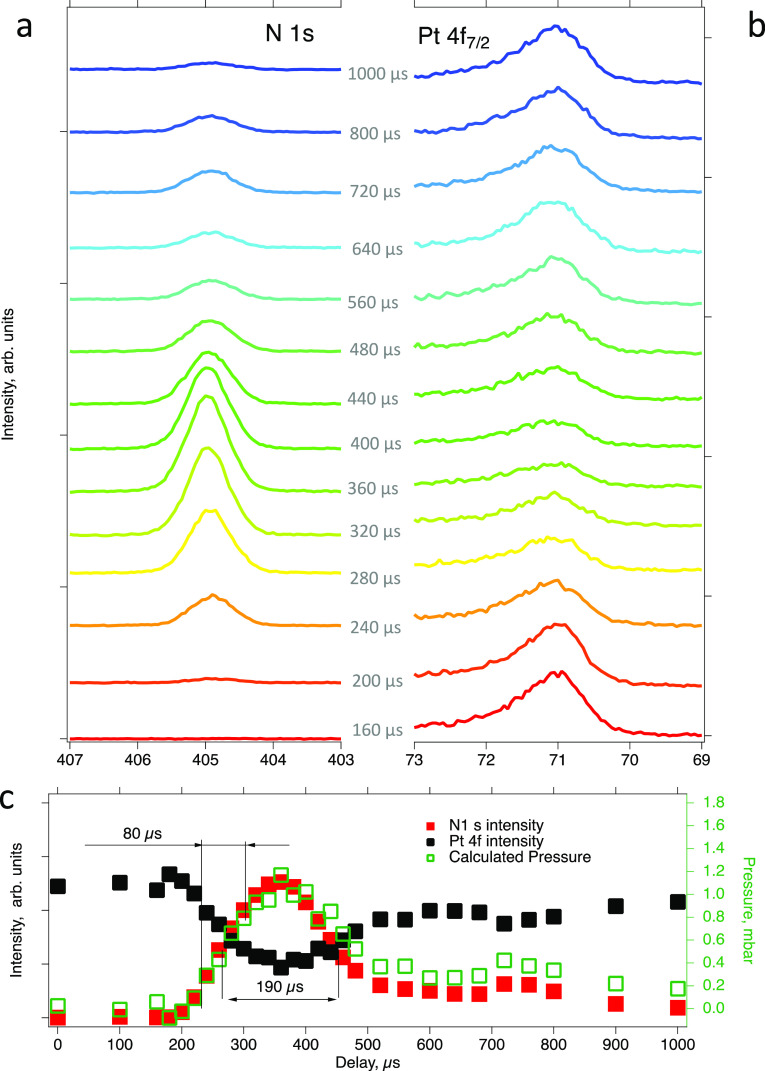
Event-averaged time-resolved XPS measured while pulsing N_2_ gas onto Pt(111). (a) and (b) show N 1s and Pt 4f_7/2_ regions after background subtraction for several time delays referenced to the N_2_ valve opening time. (c) N 1s (filled red squared) and Pt 4f_7/2_ (filled black squares) peak areas determined by curve-fitting the spectra in a and b, and the calculated pulse pressure (open green squares, see text for details) as functions of time delay. HV = 210 V, P_high-pressure side_ = 4 bar (abs), valve opening time = 250 μs, camera exposure = 40 μs, and repetition rate = 120 Hz (period = 8333 μs).

We start our presentation of the time-resolved APXPS results from characterizing short gas pulses created by the fast valve. In this first experiment, a Pt(111) sample was placed in the hybrid position, and nitrogen gas was pulsed onto its surface with a period of 8333 μs (frequency: 120 Hz) between the gas pulses. The pressure of the N_2_ gas at the high-pressure side of the fast valve was set to 4 bar (abs), and the valve opening time and applied voltage were 250 μs and 210 V, respectively. This resulted in an average chamber pressure of 1.1 × 10^–2^ mbar. Time-resolved N 1s and Pt 4f_7/2_ XP spectra were then taken with an exposure time of 40 μs. Figure 3a,b shows the N 1s and Pt 4f_7/2_ raw data for a number of time delays between 0 and 1000 μs. Both regions show a single peak, because of gas phase nitrogen in the N 1s region and metallic platinum in the Pt 4f_7/2_ region. Note the asymmetry of the peak, which is typical of metallic platinum.

The transient nature of the nitrogen gas pressure just above the sample surface is clear from inspection of the spectra from both regions. The decrease of the sample’s Pt 4f_7/2_ intensity is characteristic of the attenuation of photoelectrons in (sub)mbar pressures of gas. To quantitatively analyze the pulse duration and pressure, the N 1s and Pt 4f_7/2_ spectra were curve-fitted, from which the peak areas are plotted in Figure 3c with red and black squares, respectively. Inspection of panel c shows a 200 μs delay between valve triggering (at 0 μs) and the appearance of the rising edge of the pulse. This pulse delay is due to nitrogen gas traveling through the dosing tube. Moreover, it is clear from Figure 3c that the pulse length measured by tr-XPS is shorter than the valve duty cycle (190 μs vs 250 μs). This is due to a combination of different factors: the operation principle of the fast valve which includes decompressing an o-ring, the shape and form of the dosing pipe, and pumping speed of the experimental volume. We note that the data clearly indicate a sharp rising edge of the gas pulse. Under the given experimental conditions, it took ∼160 μs between the arrival of the gas pulse and the maximum gas concentration in the pulse. The rising edge of the gas pulse is characterized by a delay of 80 μs between reaching 20 and 80% of the pulse extremum. Furthermore, the falling edge of the pulse is long and slowly decaying. This is the result of the thin dosing pipe that elongates and smears the falling edge of the pulse in combination with the limited pumping speed inside the dosing tube and experimental cell.

Careful examination of panel c reveals a small increase in the intensity of the N 1s peak between 700 and 800 μs. This is not an experimental error, but a real signal and will be discussed below.

It is of interest to determine the pressure inside the gas pulses that arrive at the Pt surface. To do so, the inelastic mean free path of the electron in the gas is estimated from a separate experiment carried out with the sample in exactly the same place relative to the analyzer entrance (see the Supporting Information). Using this experimentally obtained IMFP, the pressure above the platinum surface was calculated from the experimentally observed attenuation of the time-resolved Pt 4f_7/2_ spectra shown in Figure 3b. The pressure is shown with green open squares in Figure 3c. The maximum pressure reaches 1 mbar followed by an extended tail of pressures in the 0.2 mbar range.

To summarize the pulse characterization experiments, we find that gas pulses with a rising edge duration of less than 100 μs and a maximum pressure in the mbar range are achievable. To the best of our knowledge, this is the first time such pulses have been created and studied with APXPS.

### 3.2 Pulse Characterization: Double-Gas Experiment

In the next section, we investigate how the presence of a constant flow of another gas influences characteristics of the gas pulses. As it was mentioned before, the presence of the second gas is important for mimicking of the real catalytic cycle and to allow for a complete reversal of the gas-pulse-induced surface transformations.

**Figure 4:**
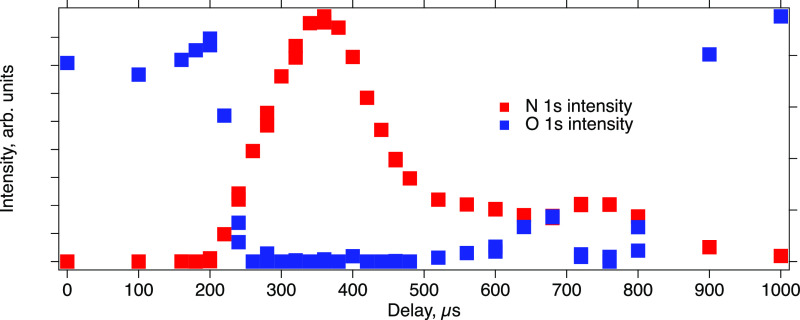
N 1s (red squares) and O 1s (blue squares) peak area evolution during N_2_ pulse into constant flow of CO_2_. Experimental parameters are the same as those in Figure 2.

In the experiment, we use a leak valve (the green component in Figure 1) to continuously dose CO_2_ gas through a thin pipe installed close to the sample surface. The background CO_2_ pressure in the chamber was 5 × 10^–3^ mbar. N_2_ was then pulsed using the fast valve with exactly the same settings on the valve and detector as in the above single-gas experiment. Only gas phase data (N 1s and O 1s) were collected, and the raw data are shown in Figure S2. The results of the curve-fitting and quantitative analysis are shown in Figure 4 with red and blue squares representing the time evolution of the N 1s and O 1s peak areas, respectively. The position, width, and shape of the nitrogen pulse mirror those of the pulse in the single-gas experiment (red squares in Figure 3c). In contrast, the signal of carbon dioxide disappears completely during the N_2_ pulse, at delays of 250 μs – 500 μs. It first reappears once the nitrogen is pumped away (after a delay of 500 μs). This is not due to attenuation of the O 1s signal by the nitrogen molecules, but rather because of the complete removal of carbon dioxide. Such behavior is expected for gases at pressures around or above 1 mbar. At these pressures, the mean free path of molecules (≪1 mm) is less than the distance between the sample surface and exit aperture of the dosing pipe (few mm), and thus the pulsed N_2_ fully displaces the CO_2_ instead of intermixing with it.

A second smaller feature can be noticed in Figure 4 at delays between 700 and 800 μs. The feature looks like another pulse of N_2_ arriving ∼500 μs after the main pulse. This feature can be attributed to the multiple reflections of the gas pulse from the surfaces in front of the sample like the analyzer cone or CO_2_ dosing tube. A similar signal in Figure 3c has the same origin because of the presence of the pipe for dosing of the second gas.

### 3.3 CO Oxidation on Pt(111)

The most intriguing feature of a use of time-resolved APXPS to monitor the interaction of a surface with a gas phase with a rapidly changing partial pressure is the potential to obtain a direct correlation between the dynamic compositions of the gas phase and surface. Photoelectron spectroscopy at mbar pressures measures not only the surface signal, but also that of the gas phase in front of the surface. It can therefore act as a time-resolved probe for reactants and products in the gas phase as well as the surface intermediates without the need for an additional gas probing technique. We demonstrate how this can be used by considering the oxidation of CO over Pt(111).

In the CO oxidation experiment, CO was pulsed into a constant O_2_ flow, which provided an O_2_ background pressure of 1.1 × 10^–2^ mbar (cf. Section 3.2 and Figure 1). The platinum sample was held at 413 °C. The fast valve (high-pressure side pressure 2.8 bar, abs.) was operating with a frequency of 40 Hz (or period of 25,000 μs), and the length of the open-close duty cycle was 200 μs. The piezo voltage was set to 280 V. The camera exposure was varied between 20 and 40 μs for different spectra.

**Figure 5:**
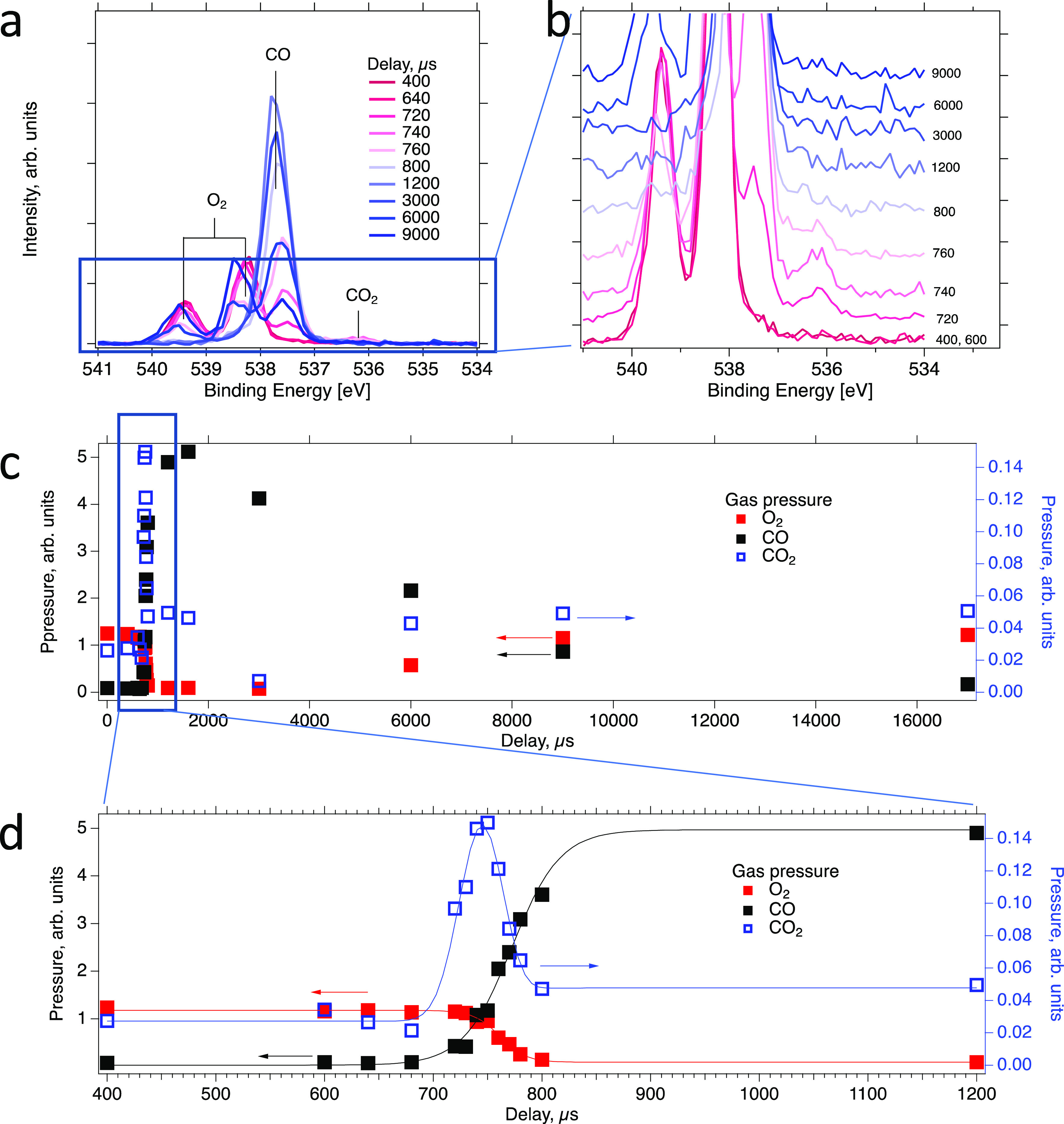
Evolution of the gas phase composition during pulsing of CO into a constant flow of O_2_ onto Pt(111). (a) O 1s gas phase spectra measured with the sample retracted by 0.3 mm from the measurement position (no surface secondary electrons are visible) for several time delays. (b) Enlarged portion of panel a with the spectra offset along the vertical axis. (c) Evolution of the O_2_ (red squares), CO (black squares), and CO_2_ (open blue squares) pressures obtained from fitting the spectra in panel a. (d) Enlarged portion of panel c.

Figure 5a shows the evolution of the gas phase with the sample in the gas phase position from a series of time-resolved O 1s spectra measured at several time delays relative to the CO pulse. A clear evolution of the O 1s spectrum is visible: At time delays between 400 and 680 μs, only two peaks are present, at 538.3 and 539.4 eV binding energy, which is characteristic of molecular oxygen.^35^ At larger delays, a clear third peak is visible at a binding energy of 537.6 eV. This feature is assigned to carbon monoxide.^35^ Its intensity evolution is inversely correlated with that of molecular oxygen.

A quantitative analysis, obtained from curve-fitting the O 1s spectra for several delays, is shown in panels in Figure 5 c and d. Red and black squares represent the intensity of the O 1s components of molecular oxygen and carbon monoxide, respectively. Panel c shows the data over a wide range of delays (0–17,000 μs), whereas panel d shows a narrower range of the same data set. Inspection of panel d shows that the transition between the O_2_ and CO atmospheres occurs in the delay range between 600 and 900 μs. As was also observed in the double-gas experiment of Section 3.2, the pulsed CO gas completely displaces the O_2_ gas within a short time. With the settings used in the experiment, the onset of the CO pulse and the decrease of the O_2_ signal coincide and happen at a delay of around 700 μs. O_2_ is displaced completely at 800 μs, which is clearly before the CO signal reaches its maximum value at 900 μs. The decrease of the oxygen signal is due to displacement by CO, whereas the increase of the CO signal is caused by the pulse pressure; as is obvious from the data their evolution may have different timescales. Here, the pressure in the CO pulse continues to rise even after it has pushed away all oxygen from the volume in between the sample and the analyzer entrance.

Turning now to the formation of CO_2_, Figure 5b shows a zoom-in on the bottom part of panel a with the spectra offset along the vertical axis. Clearly visible in this zoom-in plot is a new component at 536.3 eV binding energy, which occurs in the time delay region between 720 and 760 μs. This peak is assigned to CO_2_^35^ and clearly has a transient character. The result of curve-fitting this component is shown in panels c and d with blue open squares. Because the CO_2_ intensity is small relative to the much larger O_2_ and CO signals, we have chosen to display the signal relative to a second vertical axis, on the right-hand side of the plot.

Carbon dioxide formation starts at a delay of 720 μs. At this point, the gas phase above the surface is dominated by oxygen gas. The CO_2_ concentration reaches its maximum at a delay of 750 μs; at this point, the *P*_O_2__/*P*_CO_ ratio is 0.9. Subsequently, CO_2_ concentration decreases again and reaches its minimum value again at 800 μs, when the gas phase is dominated by CO. Interestingly, the CO_2_ signal does not reappear after 800 μs, not even at much larger time delays when the *P*_O_2__/*P*_CO_ ratio rises again. For example, the *P*_O_2__/*P*_CO_ ratio is 0.6 at a delay of 9000 μs, which is close to the ratio at maximum CO_2_ production at 800 μs. We speculate that this weakening of the CO oxidation rate probably is due to CO poisoning of the platinum surface after a long exposure to CO. It should be noted that though it is not possible to exclude the formation of a small amount of CO_2,_ it is not possible to resolve at the given signal-to-noise ratio.

Another series of O 1s spectra was measured in a similar manner to that of Figure 5. In this series, we varied the experimental parameters (valve high voltage, opening time, reaction temperature, and gas pressure on the high-pressure side). The series of individual spectra and curve-fitting results are presented in Figure S3. They are summarized in Table S1.

For all cases, several common effects are noted. First, the CO gas fully displaces the O_2_ gas from volume in front of the surface within a relatively short time (100–400 μs). Second, CO_2_ production is a fast, transient process that follows the rising edge of the CO pulse. Indeed, CO_2_ production begins when the reaction mixture is dominated by oxygen (*P*_O_2__/*P*_CO_ ≫ 0.5); it rapidly reaches a maximum rate well before reaching the stoichiometric ratio (*P*_O_2__/*P*_CO_ ratio = 0.5); and it dips to a minimum again before complete oxygen displacement. During maximum CO_2_ production, the measured *P*_O_2__/*P*_CO_ ratio was 1.9 ± 0.3, and the *P*_CO_2__:*P*_CO_ ratio reached 0.070 ± 0.008. It should be noted that for certain datasets, a small signal at around 536.3 eV binding energy, that is, the binding energy of the CO_2_ component, was still observed in the photoemission spectra at large time delays, or even before the CO pulse. This could indicate traces of residual CO that keeps being converted to CO_2_ under oxygen-rich steady-state reaction conditions. The observation is no surprise, because the pumping rate of the analysis volume is limited, the experimental volume is large, and the gas pulses have a high repetition frequency. The observed feature is, however, not particularly pronounced, and its intensity is comparable to that of the spectral noise. Therefore, we find the observation inconclusive.

**Figure 6:**
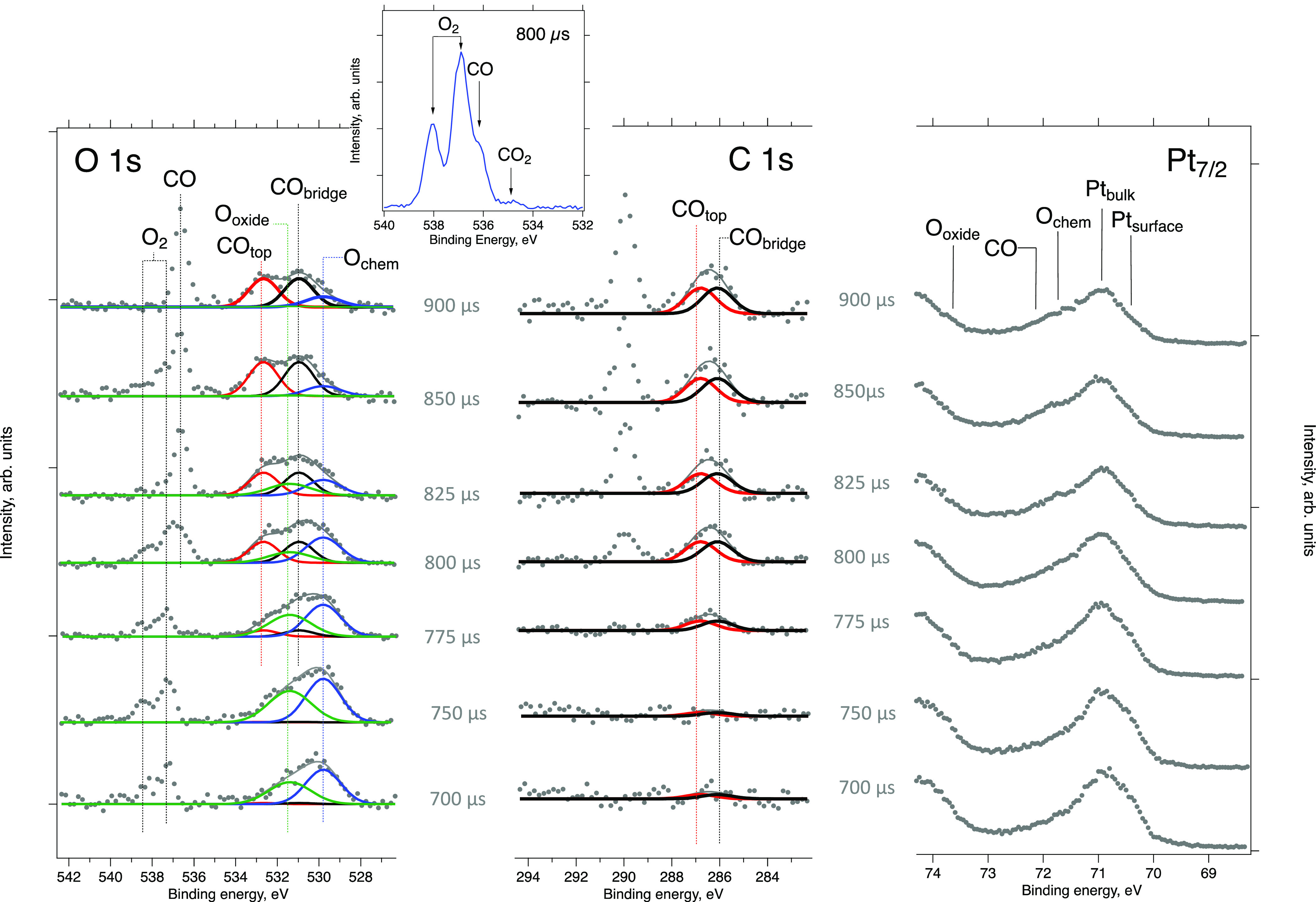
Time-resolved O 1s (left), C 1s (middle), and Pt 4f_7/2_ (right) ambient pressure photoelectron spectra recorded during pulsing of CO into a constant flow of O_2_. Inset: gas phase ambient pressure photoelectron spectra at 800 μs delay measured with the same settings as the main data set and with the sample 0.3 mm retracted from the measurement position. Note that curve-fit is intended for illustration purposes only (see details in the text). *T*_sam_ = 330 °C, detector exposure time: 80 μs, pulsing period: 30,000 μs, HV = 240 V, and *P*_high-pressure side_ = 3 bar (abs).

Finally, we consider the correlation between the evolution of the gas phase and surface composition. Figure 6 shows O 1s (left), C 1s (middle), and Pt 4f_7/2_ (right) spectra for a series of delays after CO pulsing into a constant flow of oxygen onto Pt(111). The sample was in the nominal XPS position and kept at ∼330 °C with the detector exposure time set to 80 μs. The pulsing period was set to 30,000 μs to allow for enough time for CO to pump away.

Because of the limited measurement time, it was not possible to record both the pure gas phase and surface spectra for all delays (gas phase spectra are measured with the sample retracted). It was, therefore, not possible to obtain the precise time delays for maximum gas phase CO_2_ production. Instead, we used the results from the statistical analysis shown in Figure S3 to estimate this parameter with a precision high enough for unambiguous correlation of surface and gas phase composition.

The rate of CO_2_ production could be estimated from the ratio between the dioxygen and carbon monoxide O 1s gas phase peaks (Figure 6, left). Indeed, these data clearly show a complete switch from the oxygen-dominated (delays 700 and 750 μs) to a CO-dominated (850 and 900 μs) environment. Spectra recorded at delays 775, 800, and 825 μs feature both O_2_ and CO gas phase peaks, although at different ratios. From Table S1, it is visible that the gas mixture above the surface at maximum CO_2_ production is mostly oxygen-rich (*P*_O_2__/*P*_CO_ ratio between 0.9 and 2.3 for different conditions). The corresponding delays in Figure 6 are close to 800 μs. Moreover, the *P*_CO_2__/*P*_CO_ ratio at the maximum CO_2_ production rate is between 0.053 and 0.085 (cf. Table S1). An alternative way of estimating the *P*_CO_2__/*P*_CO_ ration is from an analysis of the intensities in the O 1s gas phase spectrum recorded at 800 μs delay and under the same experimental conditions, but with the sample retracted by 300 μm (inset in Figure 6). From such an analysis, we find *P*_CO_2__/*P*_CO_ = 0.07. Altogether, it is reasonable to assume maximum CO_2_ production around 800 μs.

After establishing at which time delays the CO_2_ production rates are low and high, respectively, it becomes possible to establish a correlation between the compositions of the gas and surface phases during a CO pulse. First, a clear feature with an asymmetric shape can be observed at ∼530 eV binding energy in the O 1s spectrum (Figure 6, left) under oxygen-rich conditions (i.e., between 700 and 750 μs). This implies that at least two oxygen species are present on the platinum surface–chemisorbed oxygen and a surface oxide. The binding energies of the corresponding components were estimated to be 529.8 and 531.4 eV (see the Supporting Information for a detailed explanation of the fitting procedure), which is in good agreement with previous literature.^21−23,25,27,36−38^ C 1s spectra (Figure 6, middle), measured with the same delays, do not show any noticeable carbon signal implying a CO-free surface. Surprisingly, the Pt 4f7/2 spectrum (Figure 6, right) exhibits not only a clear bulk platinum signal at 70.9 eV, but also a shoulder at 70.5 eV that is normally assigned to adsorbate-free surface Pt atoms.^39^ Indeed, the O_chem_, CO, and O_oxide_ features in the Pt 4f region are expected at binding energies of around 71.8, 72.0, and 73.6 eV,^36−38,40,41^ that is, at higher binding energy than the bulk signal. Survey spectra measured during the experiments did not show any detectable surface contamination. The observation of a significant amount of free surface platinum atoms, despite the presence of chemisorbed oxygen and surface oxide species, is highly unexpected.

In the initial stages of the CO pulse (Figure 6, in between time delays 775 and 800 μs) new surface features develop in the O 1s region at 531.0 and 532.7 eV and C 1s region – at ∼286.1 and 286.8 eV. These new features are consistent with CO_bridge_ and CO_top_ species.^40,41^ At the same time, the intensity of the Pt 4f_7/2_ surface component starts to decrease, and a new peak appears at 72.0 eV. These new features become more prominent as the CO pulse develops, in a CO-rich environment (between time delays 850 and 900 μs). High-binding energy peaks are dominant in the O1s region; at low binding energy only a weak shoulder remains (at the same energy at which the original chemisorbed oxygen O 1s signal appeared at 700 μs time delay). In the C 1s region, the initially observed peaks reach their maximum intensity but do not change their shape. Finally, the Pt 4f_7/2_ peak surface component intensity drops to a minimum (although it does not fully disappear), whereas a shoulder at 72.0 eV reaches its maximum intensity.

**Figure 7:**
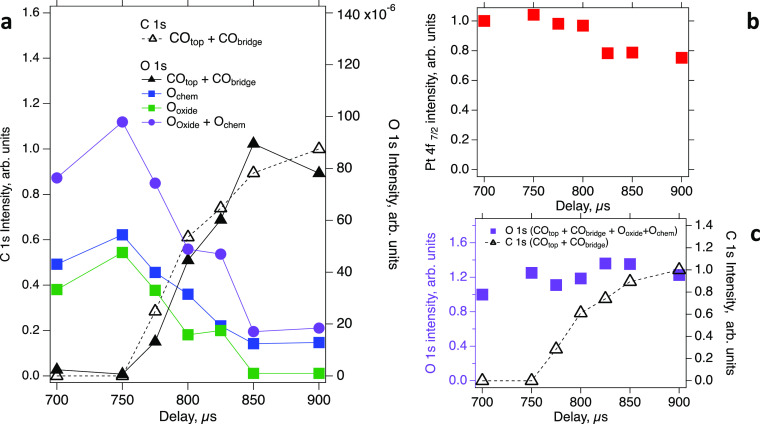
Evolution of surface components in APXPS during pulsing of CO into O_2_ obtained from curve-fitting the data in Figure 6. (a) Intensities of the C 1s peaks related to adsorbed CO (open triangles) and of the O1s peaks because of adsorbed CO (filled black triangles), chemisorbed oxygen (filled blue squares), platinum surface oxide (filled green squares), and their sum (filled purple circles) as a function of time delay. (b) Pt 4f_7/2_ intensity (no background subtracted) as a function of time delay. (c) Total O 1s (pink squares) and C 1s (open black triangles) intensities. All lines are shown for guidance.

Figure 7 presents the results of curve-fitting the data set presented in Figure 6. Panel a shows the evolution of the total C 1s intensity of both CO species (open black triangles), the sum of the O 1s intensities for both CO species (filled black triangles) and the sum of O 1s intensities for chemisorbed oxygen and oxide species (purple circles). The blue and green filled squares show the evolution of chemisorbed and oxide species, respectively. All data are normalized using the Pt 4f_7/2_ intensity to compensate for the pressure increase (see below). Panel b shows the evolution of the total Pt 4f_7/2_ intensity. It is apparent that the overall platinum signal drops by approximately 25% as the CO pulse advances, which is a signature of the elevated pressure caused by the carbon monoxide in the pulse. Finally, panel c shows the total O1s and C1s intensities of the surface species (normalized to the Pt 4f_7/2_ intensity). It is clear in panel c that the total O1s intensity of the surface species increases with time. This is quite a surprising observation because, as evident from Pt 4f_7/2_ spectra evolution (Figure 6, right), more of the platinum surface becomes occupied by adsorbed species as the CO pulse develops. This will be addressed in the discussion section.

## 4 Discussion

The observations above allow us to follow the development of the surface species during the arrival and passing of the CO pulse and to correlate them with the evolution of the gas phase. First, under oxygen-rich conditions the surface of Pt(111) is partially covered by two different oxygen species–chemisorbed oxygen and oxygen in a surface oxide—with the former being the dominant phase. As the CO pulse arrives on the surface, it interacts with O_chem_ and O_oxide_ and consumes both of them to produce CO_2_. At the maximum CO_2_ production rate (at a time delay of around 800 μs), the gas phase is dominated by oxygen; on the surface the coverage of the O species is equal to that of the CO species. Shortly after the CO_2_ production reaches minimum again (at time delays larger than 850 μs) the surface is poisoned by CO, although small amounts of chemisorbed oxygen remain on the surface even under reducing conditions.

Using the evolution of oxygen species on the surface of Pt(111) during the 100 μs-long CO_2_ production window, the turnover frequency (TOF) rate for the CO oxidation reaction can be estimated. From Figure 7a, it is clear that only 20% of the oxygen species in the surface oxide and chemisorbed oxygen remain unreacted by the end of the CO_2_ reaction window. Based on the fact that the gas phase O_2_ is rapidly replaced by the CO gas phase, we can assume that there is only a negligible increase in O surface concentration because of oxygen dissociation (cf. Figure 6). Assuming linear dependence of the oxygen species coverage from time, these results imply that on average it takes 1.25 × 10^–4^ s for O species to react with CO. Thus, the TOF rate can be estimated to be  ∼ 8 × 10^3^ conversions per active site per second, which is higher than what has been reported previously for CO oxidation over Pt (111) in the MLT.^17^ Such high values of TOFs, thus, can be an indication of the absence of mass-transfer limitations. This absence is also in agreement with a low CO_2_ conversion rate observed in all gas phase measurements. Hence, a clear advantage emerges from the time-resolved gas pulsing method as compared to steady-state averaging measurements. The latter are often performed under mass-transfer-limited conditions, which complicates the analysis of the surface reaction dynamics.

The presence of both O_chem_ and O_oxide_ during the CO_2_ production is an indication that LH and MK mechanisms together are responsible for the CO oxidation. One reason that chemisorbed oxygen remains on the surface of platinum, even under reducing conditions, could be due to a higher activity of the oxide phase. This would be in line with previous observations in which surface oxides on Pt had a high activity toward CO oxidation. Another possibility could be that the oxide phase serves as an active oxygen supply which reacts with CO. The data obtained here do not clearly favor one mechanism over the other. The main mechanism for the maximization of CO_2_ production, according to our data, is that the O/CO ratio can be kept close to 1, which occurs naturally on the surface when it is cycled between CO- and O-rich conditions.

The pulse–probe scheme presented here for obtaining timing information is relatively simple to realize when a time-gated detector such as a fast camera is used. The disadvantage of such an approach is its high inefficiency with respect to data collection. Assuming a 100 μs exposure time and 10 ms pulsing period (100 Hz), the data collection efficiency of the measurement is only 1%, that is, only 1% of the measurement time is used to collect tr data. In the remaining time, the detector idles and does not collect data. As illustrated by the measurements shown in Figure 6, the collection efficiency may be even lower: here it was only 0.27% (80 μs exposure time, 30,000 μs pulsing period). This low efficiency is the reason for a poor signal-to-noise ratio in the measured data. To collect usable data, the experimental conditions such as valve pressure, valve high voltage, gas flow, pumping rate, sample temperature, and sample–cone distance have to be as constant as possible throughout the duration of the whole experiment. Small changes in any of the conditions might affect the kinetics of the catalytic reaction or the pulse propagation characteristics, thus introducing a timing error in the measurement. In the present setup, constant experimental conditions could be guaranteed for a time length of up to 20–24 h. During this time period, the effective data acquisition time was 2.5 h–3 h for every O 1s, C 1s, and Pt 4f_7/2/_ series. Applying a 0.27% collection efficiency, the series of three spectra would take approx. 1/2 minute to record under non-time-resolved conditions. The observed spectra quality is expectable for such short acquisition times in non-time-resolved mode and could easily be compensated by a ten- or hundred-fold increase in acquisition time without significantly compromising the overall experimental time. Such an increase is of course not possible for time-resolved measurements, implying that only a handful of time delays could be measured during a single beamtime. This problem could be overcome by use of the time-sensitive event counting detectors such as the delayline detector which records with the high precision (tens of ps) time of each event. This is typically called the time recording approach. In this approach, the reference signal corresponding to the t_0_ is also recorded using the same timing source. Later all events become sorted into the time bins based on the difference between the t_0_ and following events occurring before the next t_0_. This results in obtaining ALL time delays in a single tr-XPS measurement.

## 5 Conclusions

A new method is demonstrated for generating ultrashort gas pulses (with a rising edge that is shorter 100 μs and a duration of several ms) with internal pressures in the mbar range. The ultrashort gas pulses are delivered onto a sample in an APXPS setup. The pulse generation method is combined with fast data acquisition using a hemispherical electron energy analyzer with a time-gated detector (a CCD camera with the shortest exposure time of 20 μs) to obtain time-resolved information on the evolution of the gas phase composition and the corresponding response of the solid sample’s surface. The CO oxidation reaction on Pt(111) is studied and used to demonstrate the time-, surface-, and chemical-sensitivity of the technique. The maximum CO_2_ production rate was that the TOF was 8 × 10^3^ conversions per active site per second, which is a sign of absence of the MLT during pulsing CO into a constant flow of O_2_ on Pt surface. This rate is found to be at *P*_O_2__/*P*_CO_ ratios between 0.9 and 2.3. Under these conditions, roughly equal amounts of surface CO and surface oxygen are detected. Both chemisorbed oxygen and a surface oxide are found on the Pt(111) surface. Overall, the evolution of observed species indicates that both LH and MK mechanisms play important roles in the oxidation of carbon monoxide under ambient conditions.

This is the first time that APXPS reaches a time resolution better than 1 ms in a process which uses gas perturbations at mbar internal pressure. By combining a high time resolution, chemical perturbation, and ambient conditions, the pulse–probe tr-APXPS opens new possibilities for the study of heterogeneous catalytic processes. It now becomes possible to measure not only the time-averaged, but also the time-resolved composition of solid–gas interfaces. This makes it possible to identify and follow individual reaction steps. This is a significant step toward understanding the mechanisms of catalytic reactions.

## Supporting Information Available

Details of the gas pressure calculations, individual N 1s and O 1s spectra for N_2_ pulsing into the O_2_ experiment, statistical analysis of O 1s spectra for datasets during CO oxidation on Pt(111), and description of the fitting procedure for spectra presented in Figure 6 (PDF).
