# Supplementary material for: Gas Pulse–X-Ray Probe Ambient Pressure Photoelectron Spectroscopy with Submillisecond Time Resolution
Source: ACS Appl Mater Interfaces. 2021 Sep 30;13(40):47629–41. doi: 10.1021/acsami.1c13590 (PMC8517956; doi:10.1021/acsami.1c13590)
Supplement: Supplementary file 1 — am1c13590_si_001.pdf [file am1c13590_si_001.pdf]

Supporting Information

Gas Pulse – X-ray Probe Ambient Pressure Photoelectron Spectroscopy with sub-millisecond time resolution

Andrey Shavorskiy[1], Giulio D'Acunto[2], Virginia Boix de la Cruz[2], Mattia Scardamaglia[1], Suyun Zhu[1], Robert H. Temperton[1], Joachim Schnadt[1,2], and Jan Knudsen[1,2]*

[1]MAX IV Laboratory, Lund University, Lund, 221 00, Sweden

[2]Division of Synchrotron Radiation, Department of Physics, Lund University, Lund, 221 00, Sweden

Corresponding author's email: andrey.shavorskiy@maxiv.lu.se

Gas pulse pressure calculation

To calculate the pressure within a N_2 pulse, using the data in Figure 3, the Lambert-Beer law for the attenuation of the photoelectron by the gas phase is used: $I = I_0 e^{-\frac{d}{\lambda_{gas}(P)}}$. Here, I and I_0 are the XPS intensities in the presence and absence of a gas phase, d is the distance which the electrons have to travel in the high pressure, and is the inelastic mean free path (IMFP) of the electrons in the gas phase. λ_{gas}(P) has the following dependence on the pressure: $\lambda_{gas}(P) = \frac{kT}{\sqrt{2}P\sigma_{gas}}$.[1] Combining these equations we obtain $P = C \ln\frac{I_0}{I}$, where $C =$

$\frac{kT}{d\sigma_{gas}\sqrt{2}}$ is a constant. The value for C was calculated from measuring the attenuation of the Pt 4f signal in several static pressures of gas in the mbar range with the sample in exactly same geometry as the in the experiment in the main text (Figure S1). The value of C obtained from these measurements is 1.37 $\pm$1.3 mbar.

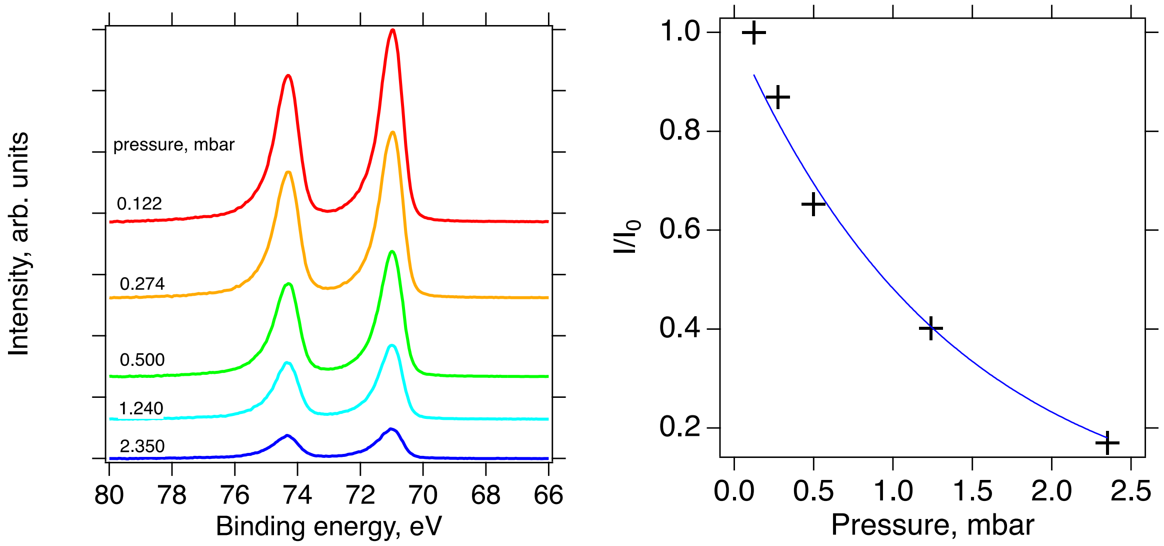

Figure S1. Static Pt 4f spectra measures at several N_2 pressures

Pulsing N_2 into CO_2

Figure S2 shows O 1s (left) and N 1s (right) time-resolved photoelectron spectra measured during pulsing N_2 gas into a constant stream of CO_2.

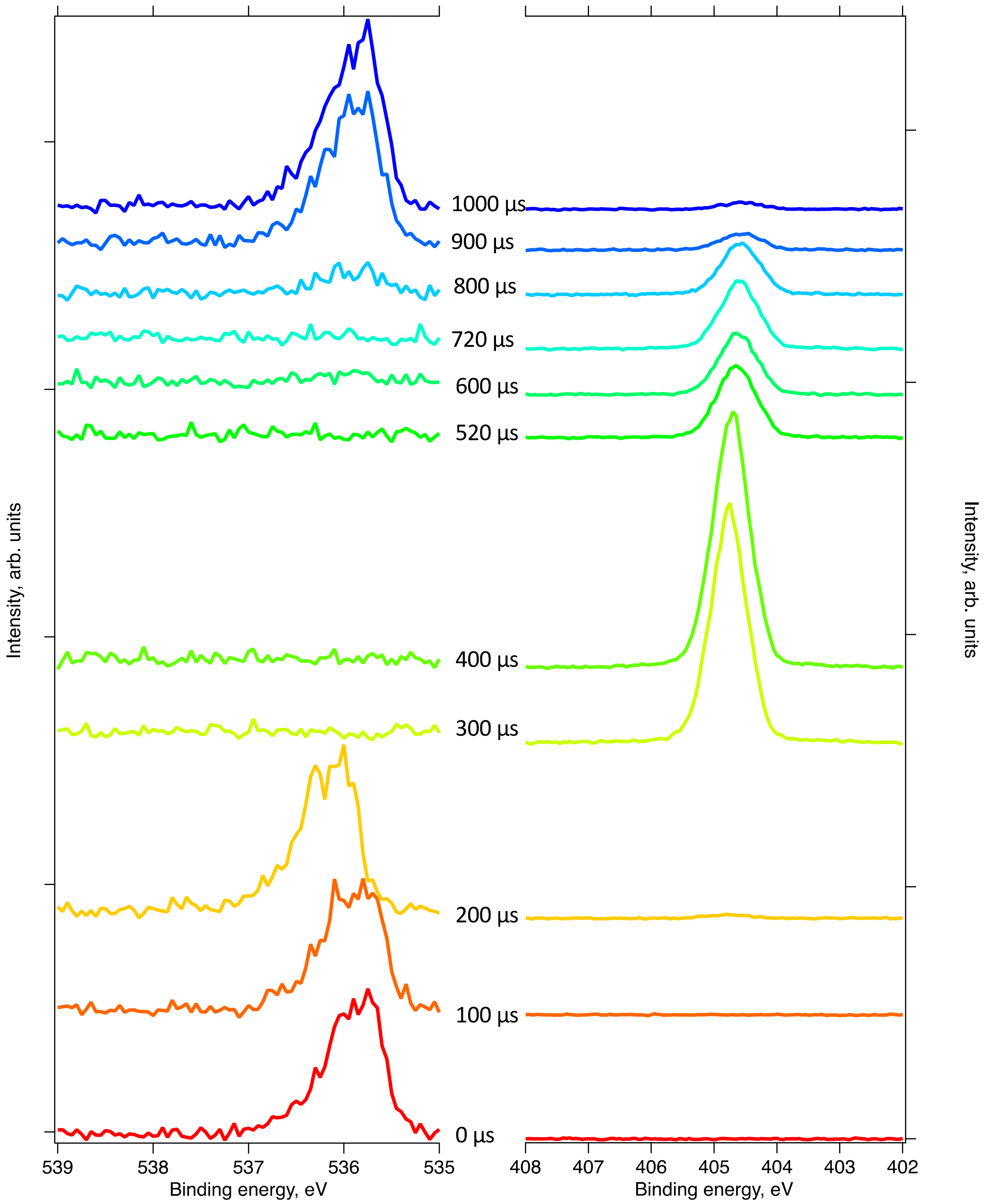

Figure S2. tr-XPS during pulsing of N_2 into a constant flow of CO_2. (left) O 1s and (right) N 1s spectra of gas phase N_2 and CO_2, respectively. The experimental conditions were the same as for the experiment the results of which are shown in Figure 4.

Time-resolved CO oxidation on Pt(111): statistical analysis

In total four datasets of O 1s gas phase spectra were measured varying experimental parameters. They were then analyzed in a manner similar to that illustrated in Figure 5. Resulting plots for are shown in Figure S3,

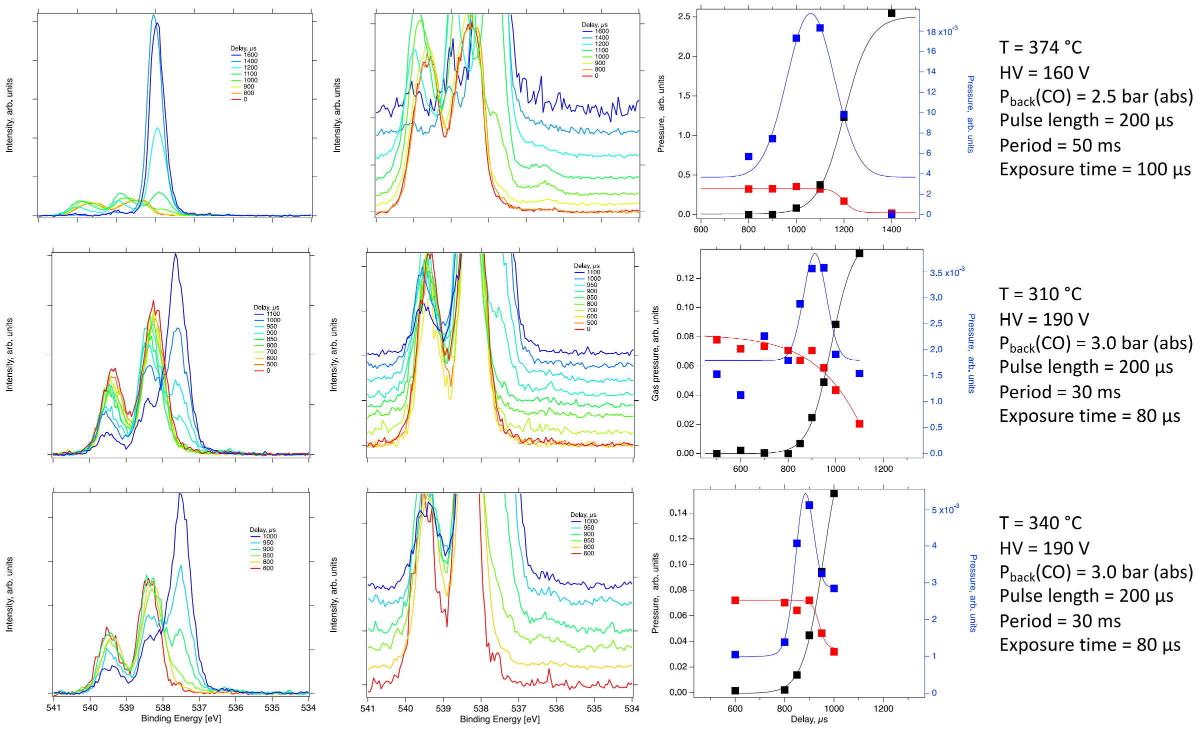

Figure S3. Evolution of the gas phase composition during pulsing of CO into a constant flow of O_2 onto Pt(111) for three different experiments (top, middle, and bottom. (left) O 1s gas phase spectra measured with the sample retracted by 0.3 mm from the XPS measurement position (no surface secondary electrons are visible) for several time delays. (middle) Enlarged portion of the left panel with the spectra offset along the vertical axis. (right) Evolution of the O_2 (red squares), CO (black squares) and CO_2 (open blue squares) pressures obtained from curve-fitting the spectra in the left panel. The experimental conditions, including the valve parameters, are depicted in the right panel.

Few effects could be clearly seen from the Figure. Thus, there is dependence of the CO rising edge and CO_2 production curve times from the pressure of CO on the high-pressure side of the

valve ($P_{back}(CO)$), which is expectable for a gas at pressures >> 1 mbar due to a low mean free path of gas molecules. Some decrease in the FWHM of the CO_2 production curve with the increase of the valve's high voltage (HV) could also be noted. However, more measurements at different HVs need to be performed to support such observation.

Despite some difference between obtained data it was possible to summarize results of all four datasets by calculating the following values: the ratio between the partial pressures of O_2 and CO at maximum CO_2 production rate, the ratio between the partial pressures of O_2 and CO at the rising edge (half-maximum) of the CO_2 production rate, the full width at half maximum of the curve related to the CO_2 production rate, and the ratio between the partial pressures of CO_2 and CO at the maximum CO_2 production rate. The values are shown in Table S1.

Table S1. Pressure ratios at characteristic CO_2 production rate parameters for CO_2 production for datasets 1-4.

set	P_{O_2}:P_{CO} @ $R_{max}^{CO_2}$	P_{O_2}:P_{CO}@ $R_{\mathrm{half}-max}^{CO_2}$	$FWHM_{CO_2}$, μs	P_{CO_2}:P_{CO} @ $R_{max}^{CO_2}$
1	0.9	2.6	50	0.067
2	2.1	12	230	0.053
3	2.2	7.0	111	0.078
4	2.3	7.1	94	0.0s85

Curve-fitting of the surface spectra in Figure 6

Assignment of the spectroscopic features in all three regions shown in Figure 6 is straightforward and is based on the components' binding energies. However, a detailed quantitative analysis and curve-fitting is complicated by (a) an insufficient quality of the data and (b) the overlapping of multiple components. Depending on the details of the initial guesses and constraints to the fit parameters the outcome of the analysis might differ strongly. Below we describe procedure which allowed us to obtain consistent fits for obtained photoemission data by simultaneously analyzing all measured regions - O 1s , C 1s , and Pt 4f – at all time

delays. Although discussed fitting results might be from the local minima, they unambiguously support conclusions of this paper.

First, we use Pt $4f_{7/2}$ spectra in Figure 6 (right) to estimate coverage of surface oxide species during measurements. These spectra clearly show Pt bulk and surface components at 70.9 eV and 70.5 eV as well as spectral components due to Pt atoms bonded to chemisorbed oxygen (O_{chem}, 71.8 eV) and carbon monoxide (72.0 eV). The absence of a prominent feature near 73.6 eV binding energy indicates only a small amount of surface oxide. Note that the strong feature at around 74 eV binding energy is the surface component of the Pt $4f_{5/2}$ peak as its intensity correlates with the intensity of the 70.5 eV Pt $4f_{7/2}$ surface feature. Thus, the Pt 4f data provides a first indication for the existence of minority oxide species under oxidative conditions.

Further, the O 1s spectra at 850 μs and 900 μs delay have clear features that, based on the literature, can be unambiguously assigned to CO_{bridge} (531.0 eV) and CO_{atop} (532.7 eV) molecules.[2,3] At the same time, the spectra at 850 μs and 900 μs delay have a shoulder at lower binding energy and cannot be fitted with just two CO peaks. This additional small intensity is assumed to originate from other oxygen species on the surface. Considering its low binding energy, these oxygen species must be of chemisorbed nature rather than surface oxide. The O 1s spectra measured at time delays of 850 μs and 900 μs thus provide a lower boundary for the binding energy and upper boundary for the full width at half maximum of the chemisorbed oxygen O 1s peak. Using this information, it is possible to narrow down the range of the fitting parameters for the chemisorbed oxygen O 1s peak in the spectra obtained at time delays of 700 μs and 750 μs. The rest of the fitting procedure involves further narrowing-down of other curve-fitting parameters from a comparison of the evolution of the O 1s and C 1s peak intensities due to CO species. If one ignores possible photoelectron diffraction effects, these two values must overlap since they originate from same species.

We used the following procedure to find optimum curve-fitting parameters for the O 1s spectra:

1. Propose new values for either the binding energy or FWHM for either the chemisorbed or oxide O 1s peak. Normally, proposed parameter was then fixed, and the rest were allowed to be fitted in step 2. For simplicity, both the chemisorbed oxygen and oxide peaks were assumed to have symmetric Gaussian shapes.

2. Curve-fit spectra at 700 μs, 750 μs, 850 μs, and 900 μs delays using the proposed parameters. If the new parameter values led to better fitting results (goodness of the fit was assessed by the chi-square test[4]) than current values, make them current and move to step 3. Otherwise, go back to step 1.

3. Using the fixed parameters, curve-fit all spectra in the series and assess the goodness of the fit in the same way as in the step 2. If it provides satisfactory results within the level of the data noise, move to step 4. Otherwise go to step 1.

4. Calculate the sum of intensities for the CO species and compare its evolution with the one obtained from the evolution of the corresponding components in the C 1s spectra.

This procedure was manually repeated using 0.1 eV binding energy and FWHM steps until a minimum difference between the O 1s and C 1s components for CO was obtained. The resulting curve-fits are shown in Figure 6 (left).

References

---

1 Ogletree, D. F.; Bluhm, H.; Lebedev, G.; Fadley, C. S.; Hussain, Z.; Salmeron, M. A Differentially Pumped Electrostatic Lens System for Photoemission Studies in the Millibar Range. Review of Scientific Instruments 2002, 73 (11), 3872–3877.

2 Toyoshima, R.; Yoshida, M.; Monya, Y.; Suzuki, K.; Amemiya, K.; Mase, K.; Mun, B. S.; Kondoh, H. A High-Pressure-Induced Dense CO Overlayer on a Pt(111) Surface: A Chemical Analysis Using in Situ near Ambient Pressure XPS. Phys. Chem. Chem. Phys. 2014, 16 (43), 23564–23567.

3 Björneholm, O.; Nilsson, A.; Tillborg, H.; Bennich, P.; Sandell, A.; Hernnäs, B.; Puglia, C.; Mårtensson, N. Overlayer Structure from Adsorbate and Substrate Core Level Binding Energy Shifts: CO, CCH3 and O on Pt(111). Surface Science 1994, 315 (1–2), L983–L989.

4 Curve Fitting. https://www.wavemetrics.com/products/igorpro/dataanalysis/curvefitting Accessed 14 Sep. 2021
